# How to Convince Clinicians that ‘Soft’ Skills Save Lives? Practical Tips to Use Clinical Studies to Teach Physicians’ Roles

**DOI:** 10.15694/mep.2019.000119.1

**Published:** 2019-05-30

**Authors:** Alexandre Lafleur, Mathieu Gagné, Véronique Paquin, Claudie Michaud-Couture

**Affiliations:** 1 Faculty of Medicine

**Keywords:** Non-medical expert roles, Clinical skills, CanMEDS Intrinsic roles, competency-based medical education

## Abstract

This article was migrated. The article was marked as recommended.

The implementation of competency-based medical education is hampered by unsupported arguments like ‘soft’ skills are important, but they don’t save lives. When implementing teaching and assessment methods targeting non-medical expert roles, student and physician buy-in is crucial. These intrinsic roles (e.g. collaborator or professional) are unfortunately misinterpreted and underused by supervisors, in part because of the false assumption that those skills have minimal impact on patient outcomes. On the contrary, although not worded in those terms, many clinical studies prove the impact of those roles on patient mortality, morbidity, readmission rate, or compliance. Whereas physicians feel that they are properly trained to give feedback, they struggle in making this connection between clinical studies and intrinsic roles in their everyday teaching habits. In this article, we provide *practical tips* on why and how to use high-impact clinical studies to enlighten supervisors and trainees about the educational and clinical importance of those skills. A slide kit, to be presented in clinical settings, provides a selection of 30 examples of ‘hard’ evidence on those so-called ‘soft’ skills, reinforcing the fact that intrinsic roles are intertwined with the medical expert role to improve patient care.

## Introduction

The implementation of competency-based medical education (CBME) is hampered by unsupported arguments like ‘soft’ skills are important, but they don’t save lives ( Van Luijk et al., 2012). Student and physician buy-in is crucial when implementing teaching and assessment methods targeting all the roles played by physicians ( Dath and Iobst, 2010). Trainees must develop the clinical skills that combine their role of medical expert with the intrinsic roles of physicians (e.g. collaborator or professional) to provide high quality care ( Sherbino et al., 2011; Steven et al., 2014; Frank et al., 2015).

Facing decisive choices, teaching the medical expert role can monopolize an unbalanced amount of time and resources on the false assumption that intrinsic roles have less impact on patient outcome ( Roberts et al., 2013; Renting et al., 2017; Schmid et al., 2017). On the contrary, although not worded in those terms, many clinical studies prove the impact of all the roles played by physicians on patient mortality, morbidity, readmission rate, or compliance (examples presented in Tables 1 to 6). Physicians however struggle in making the connection between clinical topics and intrinsic roles ( Roberts et al., 2013; Renting et al., 2016; Renting et al., 2017).

We wrote this article in the *practical tip* format to explain why and how educators can use a selection of high-impact clinical studies to exemplify the intrinsic roles of physicians for their colleagues and trainees during the transition to CBME. It will underpin the educational and clinical importance of giving feedback on intrinsic roles in daily activities, focusing on interventions with proven clinical impact, e.g. referral and counselling for smoking cessation, multidisciplinary teamwork for chronic diseases, quality of handoffs in acute care settings, quality of follow-up on laboratory results, etc. ( Hassan et al., 2015).

We selected thirty studies to cover a broad range of clinical topics and disciplines. They are categorized in Tables 1 to 6 with respect to the intrinsic role predominantly involved. We chose the CanMEDS framework because of its comprehensiveness and evidence in the medical education literature supporting our problem statement ( Sherbino et al., 2011; Frank et al., 2015). The definitions and keywords provided in Table 7 allows readers to make links with other competency frameworks in health sciences education ( Englander et al., 2013).

By explaining our method of selection and categorization, our intent is that clinical teachers use these references, and others that they can find, to fuel their daily feedback and discussions with trainees. A slide kit, ready to be presented in clinical settings, provides a visual summary for each study (see PowerPoint© supplementary file). We believe it will reinforce the fact that, far from being an abstract construct, intrinsic roles are intertwined with the medical expert role in many successful clinical interventions.

### Intrinsic roles still misinterpreted and underused

In the CanMEDS framework, non-medical expert roles (i.e., communicator, collaborator, manager, scholar, professional, and health advocate) are better named intrinsic roles ( Sherbino et al., 2011; Frank et al., 2015).Although most clinical teachers are familiar with the CanMEDS roles or other competency frameworks, discourse analysis of the feedback given in the workplace shows that physicians often misinterpret the meaning of intrinsic roles ( Renting et al., 2016). Feedback on efficiency, directive leadership, and resource management predominates. Patient-centered and teamwork approaches are at the essence of those roles, yet they are rarely discussed with students ( Renting et al., 2016). Observations in clinical settings show that the roles are rarely explicitly named or used to structure daily interactions between residents and physicians ( Renting et al., 2017). For example, in 2010, less than 50% of urology residents reported that the communicator role had been targeted by formal teaching or feedback by supervisors ( Roberts et al., 2013).

### Clinician buy-in is a prerequisite for competency-based medical education

As summarized by experts, “[CBME] requires front-line medical teachers to understand, accept, teach, and evaluate domains of practice beyond medical expertise” ( Dath and Iobst, 2010). For learning to occur after a patient encounter, the clinical teacher must reinforce intrinsic roles through feedback and supportive dialogue ( Steven et al., 2014). When implementing a post-graduate CBME curricula in the Netherlands, the implementation of the intrinsic roles in the teaching habits of physicians was identified as a major barrier ( Van Luijk et al., 2012). As a result, although graduates are comfortable in their medical expert role, they feel inadequately prepared in their manager, leader, and communicator roles ( Schmid et al., 2017). Preparedness scores are lowest for tasks concerning management administration and leadership, research, end-of-life care, and safety-related patient communication( Schmid et al., 2017).

### Patient outcome: the ultimate argument to influence teaching habits

It will take efforts from clinical teachers to increase the exposure of trainees to clinical situations in which an intrinsic role is actively involved. As observed by Renting, Raat, Dornan, et al., the clinical tasks assigned to residents rarely allow them to learn the health advocate and leader roles ( Renting et al., 2017). Although professional and communicator roles are often involved, all other roles total less than 20% of clinical activities and are scarcely observed by supervisors ( Schmid et al., 2017).

Leaders in medical education have the power to inform and motivate their colleagues. Their arguments should target the main reasons why physicians rarely teach intrinsic roles, in particular health advocate manager, scholar, and professional: lack of time, lack of interest, and the misbelief that these roles cannot be taught ( Arora et al., 2009; Whitehead et al., 2011). Although physicians feel that they are properly trained to give feedback, they struggle in making the connection between clinical topics and intrinsic roles ( Van Luijk et al., 2012). We believe that associating intrinsic roles with clinical indicators of performance can be an incentive for clinicians who usually focus their teaching and feedback on interventions with proven clinical impacts ( Landon et al., 2003).

## Exemplifying intrinsic roles with high-impact clinical studies

### Collaborator

As seen in Table 1, working effectively with other professionals translates into better patient care (i.e. reduced mortality, infection rates, rehospitalization, medical errors, and adverse events). Most high-impact studies describe a structure of co-management by healthcare professionals (interprofessional or multidisciplinary) or a procedure for a complete and efficient transfer of clinical information (e.g., handovers) ( McAlister *et al.*, 2004; Friedman *et al.*, 2009; Kim *et al.*, 2010; Neily *et al.*, 2010; Starmer *et al.*, 2013). The studies demonstrating a high impact on mortality or morbidity target ‘at-risk’ patients in intensive care, post-operative care, and ambulatory clinics for chronic diseases, or patients transferring to another care unit.

**Table 1.** Selection of Clinical Studies Exemplifying the Impact of Collaboration Skills

Selected studies	Interventions	Participants	Type of study and methods	Clinical outcomes
Association between implementation of a medical team training program and surgical mortality (Neily *et al*., 2010)	Team training program (briefings and debriefings in the operating room): 2 months of preparation, 1-day conference, 1 year of quarterly coaching interviews	182 409 sampled procedures from 108 facilities that provided surgical care to veterans	Retrospective health services cohort study using a contemporaneous control group	The 74 facilities in the training program experienced an 18% reduction in annual mortality compared with a 7% decrease among the 34 facilities that had not yet undergone training.
Impact of a comanaged Geriatric Fracture Center on short-term hip fracture outcomes (Friedman *et al*., 2009)	Patients are comanaged daily by a geriatrician and orthopedic surgeon, emphasizing total quality management, timely treatment, and standardized care.	314 patients aged 60 years or more in 2 hospitals in Rochester, NY	Retrospective cohort study	Compared with usual care, comanaged patients had shorter times to surgery (24.1 vs 37.4 hours), fewer postoperative infections (2.3% vs 19.8%), fewer complications overall (30.6% vs 46.3%), and shorter length of stay (4.6 vs 8.3 days).
Multidisciplinary strategies for the management of heart failure patients at high risk for admission (McAlister *et al.,* 2004)	Outpatient-based multidisciplinary management strategies in HF: specialized multidisciplinary team, telephone follow-up, educational programs	29 trials (5,039 patients)	Systematic review of randomized trials of multidisciplinary management programs in HF	27% reduction in HF hospitalization rates and 43% reduction in total number of HF hospitalizations. Follow-up by a specialized multidisciplinary team reduced all-cause mortality by 25%.
Rates of medical errors and preventable adverse events among hospitalized children following implementation of a resident handoff bundle (Starmer *et al*., 2013)	Resident handoff bundle: standardized communication and handoff training, verbal mnemonic and new team handoff structure.	1255 patient admissions involving 84 resident physicians on 2 inpatient units at Boston Children’s Hospital	Prospective intervention study	The rates of medical errors decreased from 33.8 to 18.3 per 100 admissions and preventable adverse events measured decreased from 3.3 to 1.5 per 100 admissions, following the intervention.
The effect of multidisciplinary care teams on intensive care unit mortality (Kim *et al*., 2010)	Daily rounds by a multidisciplinary care team	107 324 patients admitted to 112 acute care hospitals in Pennsylvania	Population-based retrospective cohort study, linking a statewide hospital organizational survey to hospital discharge data	ICU multidisciplinary care was associated with significant reductions in the odds of death (OR 0.84, CI 0.76-0.93).

### Communicator

Communication skills will make a difference in the outcome of patients, especially if they are in a state of vulnerability, including being under stress, suffering from mental illnesses, being hospitalized, facing a terminal illness, or having under-developed literacy skills. In these populations, the quality of communication between the clinician and his or her patients (or relatives) showed benefits in the indicators of morbidity (e.g., glycemic control), understanding of and compliance with treatments, readmission rate, and quality of end-of-life care ( Schillinger *et al.*, 2003; Arbuthnott and Sharpe, 2009; Detering *et al.*, 2010; Légaré *et al.*, 2012; Carter *et al.*, 2018). Selected examples can be found in Table 2.

**Table 2.** Selection of Clinical Studies Exemplifying the Impact of Communication Skills

Selected studies	Interventions	Participants	Type of study and methods	Clinical outcomes
Closing the loop: physician communication with diabetic patients who have low health literacy (Schillinger *et al*., 2003)	Physicians assessment of recall and comprehension of new concepts	38 physicians and 74 patients with diabetes mellitus and low functional health literacy	Observational prospective study using audiotapes of visits	Patients whose physicians assessed recall or comprehension were more likely to have lower HbA1c.
The effect of physician-patient collaboration on patient adherence in non-psychiatric medicine (Arbuthnott and Sharpe, 2009)	Physician-patient collaboration variables (any aspect of physician-patient communication, interaction, participation, satisfaction with level of involvement, education, decision-making, and alliance)	34,000 non psychiatric participants in 48 studies	Meta-analysis	A statistically significant weighted mean effect size indicated better physician-patient collaboration is associated with better patient adherence.
The association between patient experience factors and likelihood of 30-day readmission (Carter *et al*., 2018)	Patient perceptions of care (confidence in ability to perform self-care after discharge, satisfaction with inpatient care received, understanding of the care plan)	846 patients admitted to 2 inpatient medical units at the Massachusetts General Hospital	Prospective cohort study using interviewer-administered surveys	Respondents who reported being ‘very satisfied’ with the care received during the index hospitalisation were less likely to be readmitted (aOR 0.61, 95% CI 0.43 to 0.88). Those reporting doctors ‘always listened to them carefully’ were less likely to be readmitted (aOR 0.68, 95% CI 0.48 to 0.97).
The impact of advance care planning on end of life care in elderly patients ( Detering *et al.,*2010)	Facilitated advance care planning (aimed to assist patients to reflect on their goals, values, and beliefs; to consider future medical treatment preferences; to appoint a surrogate; and to document their wishes)	309 legally competent medical inpatients aged 80 or more in a university hospital in Melbourne, Australia	Prospective randomised controlled trial	End of life wishes were much more likely to be known and followed in the intervention group (86%) compared with the control group (30%). Family members of patients who died had significantly less stress, anxiety and depression with advance care planning.
Training family physicians in shared decision-making to reduce the overuse of antibiotics in acute respiratory infections ( Légaré *et al*., 2012)	Training program in shared decision-making (DECISION2+): 2-hour online tutorial followed by a 2-hour on-site interactive workshop.	359 patients with a diagnosis of acute respiratory infection who consulted 149 physicians in 9 family practice teaching units in 6 regions of Quebec	Multicentre, parallel cluster randomized trial. The trial had 2 arms and was conducted in 3 phases: baseline data collection, intervention and postintervention data collection.	DECISION+2 enhanced patient participation in decision-making and led to fewer patients deciding to use antibiotics (27.2% vs 52.2%, aRR 0.48, 95% IC 0.34-0.68).

### Scholar

The scholar role certainly overlaps with other intrinsic roles. Nevertheless, studying the adherence to practice guidelines is directly related to the translation of new knowledge into clinical practice. Table 3 presents studies in which commitment to excellence in practice through professional development lead to improvement in hospitalization rates, morbidity, and survival ( Davis *et al.*, 1999; Mallett *et al.*, 2000; Le Pen *et al.*, 2005; Menéndez *et al.*, 2005; Rodríguez *et al.*, 2005). A higher impact of continuing medical education is seen when active learning occurs through interactive sessions and personal practice audits.

**Table 3.** Selection of Clinical Studies Exemplifying the Impact of Scholar Skills

Selected studies	Interventions	Participants	Type of study and methods	Clinical outcomes
Adherence to guidelines is a predictor of outcome in chronic heart failure ( Le Pen *et al.,* 2005)	Physicians’ adherence to European Society of Cardiology guidelines for heart failure medication	1410 patients from France, Germany, Italy, Netherlands, Spain, and UK. 150 cardiologists were randomly selected.	Observational study	Adherence of physicians to treatment guidelines is a strong predictor of fewer CHF and cardiovascular hospitalizations. CHF and CV hospitalization rates were, respectively, 6.7, 9.7, and 14.7% and 11.2, 15.9, and 20.6% for perfect, moderate and low adherence.
Antibiotic Prescription for Community-Acquired Pneumonia in the Intensive Care Unit: Impact of Adherence to Infectious Diseases Society of America Guidelines on Survival ( Rodríguez *et al*., 2005)	Adherence to the Infectious Diseases Society of America guidelines	529 patients with severe CAP in 33 Spanish hospitals.	Prospective multicenter study	Higher mortality was documented among patients with nonadherence to treatment guidelines (33.2% vs. 24.2%).
Guidelines for the Treatment of Community-acquired Pneumonia (Menéndez *et al*., 2005)	Adherence to the Spanish guidelines for the empiric antibiotic treatment of CAP	1,288 patients with CAP admitted to 13 Spanish hospitals.	Multicenter observational prospective study	Adherence to the guidelines was found protective for mortality (OR 0.55; 95% CI 0.3-0.9) and for treatment failure (OR, 0.65; 95% CI, 0.5-0.9).
Impact of Formal Continuing Medical Education: Do Conferences, Workshops, Rounds, and Other Traditional Continuing Education Activities Change Physician Behavior or Health Care Outcomes?( Davis *et al*., 1999)	Formal continuing medical education (CME) interventions (such as conferences, rounds, meetings, symposia, and individualized training sessions)	14 randomized controlled trials of 17 formal educational interventions	Systematic review	Interactive CME sessions can effect change in professional practice (standardized effect size, 0.67; 95% CI, 0.01-1.45) and, on occasion, health care outcomes.
Reducing red blood cell transfusion in elective surgical patients: the role of audit and practice guidelines ( Mallett *et al.,* 2000)	Guidelines for peri-operative red cell transfusion	3554 elective surgical patients in a large teaching hospital in London, UK.	Two prospective 3-month audits in 1996 and 1998 (before and 18 months after the guidelines had been established)	43% reduction in the total number of transfusions over the two periods studied.

### Health advocate

Advocating to improve the health of communities or at-risk populations is a role a physician plays everyday in his or her clinical practice and at times within the public domain. Most high-impact studies describe simple measures of preventive medicine that trainees can implement in their daily practice. Exercise prescription, tobacco cessation, vaccination, and cancer screening are some of the examples detailed in Table 4 ( Nichol *et al.*, 1994; Petrella *et al.*, 2003; Anthonisen *et al.*, 2005; Fenton *et al.*, 2011; O’connor *et al.*, 2014). In fact, the ability to provide tailored counselling and follow-up leads to a reduction in illness rates for the most common and deadly diseases of our societies.

**Table 4.** Selection of Clinical Studies Exemplifying the Impact of Health Advocacy Skills

Selected studies	Interventions	Participants	Type of study and methods	Clinical outcomes
Can primary care doctors prescribe exercise to improve fitness? The step test exercise prescription (STEP) project (Petrella *et al*., 2003)	STEP included exercise counseling to physicians and prescription of an exercise training heart rate (step test: stepping up and down 2 steps 20 times). Physicians in the control group were asked to do exercise counseling and prescription as usual.	284 healthy community-dwelling patients aged 65 years+ from 4 academic primary care practices, recruited in 1998 -1999.	Randomized controlled trial. Baseline assessment and intervention delivery with post intervention follow-up at 3, 6, and 12 months.	VO2max was significantly increased in the STEP group. Patients in the STEP group reported better exercise self-efficiency. A reduction in systolic blood pressure and in BMI was noted in patients of the STEP group.
Behavioral Sexual Risk-Reduction Counseling in Primary Care to Prevent Sexually Transmitted Infections: A Systematic Review (O’connor *et al.,* 2014)	Heterogeneous interventions between studies ranging from brief individual meetings with a counselor to group sessions with educative or behavior change components.	31 trials conducted in adolescents or adults. Most studies with high-risk populations for STIs.	Systematic review.Study selection, rating of studies regarding methodology. Analyses stratified by age and intervention intensity.	High-intensity (>2 hours) interventions were likely to reduce the rate of STIs in both in adolescents (odds ratio, 0.38 [95% CI, 0.24 to 0.60]) and adults (odds ratio, 0.70 [CI, 0.56 to 0.87]).
The efficacy and cost effectiveness of vaccination against influenza among elderly persons living in the community(Nichol *et al.,* 1994)	Observation of vaccination rates, mortality and complications for each cohort.	Over 25 000 patients in each cohort (one for three consecutive influenza seasons). Adults aged of 65 years or older.	Serial cohort study.Data collection from databases.	Vaccination against influenza is associated with reduced rates of hospitalization and in deaths from influenza and its complications.
Physician Counseling for Colorectal Cancer Screening: Impact on Patient Attitudes, Beliefs, and Behavior(Fenton *et al*., 2011)	Patients completed validated measures of behavioral constructs associated with CRC screening (before and after visits). Audio-recorded discussions were analyzed. 6 months follow-up was done to verify if screening was ordered and completed.	50 patients and 20 primary care clinicians in 2 academic primary care clinics.	Prospective cohort study. Descriptive analyses to characterize patients and CRC screening conversations. Statistical analyses to verify effects of these conversations.	CRC screening discussion was associated with increased perceived risk and susceptibility to CRC, advancement in intention to get screening and completion of screening. Behavioral constructs were not associated with increased motivation to get screening.
The Effects of a Smoking Cessation Intervention on 14.5-Year Mortality: A Randomized Clinical Trial (Anthonisen *et al.*, 2005)	10-week smoking cessation program including a strong physician message and 12 group sessions using behavior modification and nicotine gum, and ipratropium or a placebo inhaler.	5887 middle-aged volunteers with asymptomatic airway obstruction.10 clinical centers in the USA and Canada.	Randomized controlled trial. Special intervention participants received the smoking intervention program and were compared with usual care participants.	At 5 years, 21.7% of special intervention participants stopped smoking (vs 5.4% with usual care). All-cause mortality was significantly lower in the special intervention group (8.83 vs. 10.38 per 1000 person-years).

### Leader

Physicians have a responsibility regarding the delivery of high-quality care in their hospital, community, and healthcare system. Coordinating an effective team, acting as opinion leader, or conducting a stewardship program improves the quality of care and patient safety. In the selected studies showed in Table 5, physicians’ leadership roles are explicitly mentioned as a key element in the success of a concerted effort ( Weingarten *et al.*, 1993; Young *et al.*, 1997; Strasser *et al.*, 2005; Blackwood *et al.*, 2011; Malani *et al.*, 2013). Some medical interventions, like the example of ventilation weaning, do not succeed unless the treating physician has the leadership skills to educate and motivate his or her team toward a common goal.

**Table 5.** Selection of Clinical Studies Exemplifying the Impact of Leadership Skills

Selected studies	Interventions	Participants	Type of study and methods	Clinical outcomes
Best Practices for Managing Surgical Services: The Role of Coordination (Young *et al*., 1997)	Examination of the role of coordination in surgical services as a determinant of patient outcomes. First phase: clinical and outcome data obtained. Second phase: survey of surgical staff of 44 services and on-site visits of 20 surgical services.	Approximately 87,000 patients from 44 surgical services of Department of Veterans Affairs from October 1991 to December 1993.	Prospective observational study. Data collection of coordination practices in the different surgical services and comparing them in relation to their mortality and morbidity outcomes.	Comparing low and high outliers (in regards to mortality or morbidity), low outliers used a greater number and a greater variety of coordination practices for each of the three work activities studied.
Clinical and economic outcomes from a community hospital’s antimicrobial stewardship program (Malani *et al*., 2013)	Audition of new starts and weekly use of 8 target antimicrobials. Description of clinical and economic outcomes from the first year of their hospital’s ASP (antimicrobial stewardship program).	510 antimicrobial orders were reviewed during the first year of the program.	Retrospective observational study. Comparison of outcomes from the first year of the program with outcomes from a similar period before institution of the program.	Implementation of the ASP was associated with an approximate 50% reduction in the odds of developing C. difficile. A decrease in defined daily doses of target antimicrobials and in pharmacy costs were observed.
Reducing lengths of stay for patients hospitalized with chest pain using medical practice guidelines and opinion leaders (Weingarten *et al*., 1993)	Education of physicians about practice guidelines to promote shorter lengths of stay (LOS) for “low-risk” patients hospitalized with chest pain and data collection of LOS.	208 patients with low-risk chest pain	Controlled-interventional trial. Measurement of patient outcomes and lengths of stay before and after implementation of the practice guidelines.	Total hospital (-22%) and intermediate care unit (-17%) lengths of stay were significantly reduced.
Team Functioning and Patient Outcomes in Stroke Rehabilitation (Strasser *et al*., 2005)	Data collection of team member perceptions of team functioning and primary patient variables (functioning improvement, discharge home and length of rehabilitation stay).	46 Veterans Administration rehabilitation teams, including 530 rehabilitation team members from 6 disciplines. 1688 stroke patients.	Prospective observational study. Evaluation of the relationship between rehabilitation team functioning and stroke patient outcomes.	3 of the 10 measures of team functioning were significantly associated with patient functional improvement (task orientation, utility of quality information and order and organization).
Use of weaning protocols for reducing duration of mechanical ventilation in critically ill adult patients: Cochrane systematic review and meta-analysis (Blackwood *et al.,* 2011)	Measurement of the effects of weaning protocols on the total duration of mechanical ventilation, mortality, adverse events, quality of life, weaning duration, and length of stay in the ICU and hospital.	11 trials that included 1971 patients.	Systematic review with meta-analysis. Randomized and quasi randomized controlled trials of weaning from mechanical ventilation with or without protocols.	Reduction in the duration of mechanical ventilation, weaning, and stay in the intensive care unit when standardised weaning protocols are used, although there is heterogenicity in the studies.

### Professional

The professional role is revealed through an ethical practice, high personal standards of behaviour, and accountability to the profession and society. How physicians follow up with patients and laboratory results is a common theme in the literature and has a significant impact on readmission rates and delays in diagnoses and treatments ( Nelson, Maruish and Axler, 2000; Callen *et al.*, 2012). Physicians’ behaviour and even their attire will influence patient confidence and lead to better compliance with pharmacological and non-pharmacological treatments ( Pronovost *et al.*, 2002; Rehman *et al.*, 2005; Canale *et al.*, 2012). Table 6 details those examples.

**Table 6.** Selection of Clinical Studies Exemplifying the Impact of Professional Skills

Selected studies	Interventions	Participants	Type of study and methods	Clinical outcomes
Effects of Discharge Planning and Compliance with Outpatient Appointments on Readmission Rates (Nelson, Maruish and Axler, 2000)	United Behavioral Health of Georgia (UBH-GA) encouraged inpatient facilities to ensure follow-up after discharge and verifies with outpatient facilities if one appointment was kept.	During 1998, 3113 psychiatric admissions in eight southeastern states, 542 were readmissions.	Prospective cohort study.Rehospitalization rates were calculated at 90, 180, 270 and 365 days after discharge to examine effects over time.	Patients who did not have an outpatient appointment after discharge were two times more likely to be re-hospitalized in the same year.
Failure to Follow-Up Test Results for Ambulatory Patients: A Systematic Review (Callen *et al.,* 2012)	Review of evidence quantifying the extent of failure to follow-up test results and the impact for ambulatory patients.	19 articles were eligible. All studies were conducted in the United States between 1995 and 2010.	Systematic review.Selection of studies with quantitative evidence of the number of tests not followed up for patients attending ambulatory settings.	Rates of tests not followed-up varied between studies. The impact on patients were missed cancer diagnoses, increased visits to hospitals to name a few.
Physician Staffing Patterns and Clinical Outcomes in Critically Ill Patients: A Systematic Review (Pronovost *et al*., 2002)	Evaluation of the association between ICU physician staffing (high-intensity characterized by an intensivist consultation or closed ICU) and patient mortality and length of stay (LOS).	26 observational studies between January 1965 and December 2001	Systematic review.Selection of trials studying ICU attending physician staffing strategies and the outcomes of hospital and ICU mortality and length of stay.	High-intensity vs low-intensity ICU physician staffing is associated with reduced hospital and ICU mortality and length of stay.
The Relationship Between Physician Empathy and Disease Complications: An Empirical Study of Primary Care Physicians and Their Diabetic Patients in Parma, Italy (Canale *et al*., 2012)	Participating physicians completed a Jefferson Scale of Empathy form. Measurement of acute metabolic complications using ICD-9-CM codes for patients hospitalized in 2009.	20,961 patients with type 1 or type 2 diabetes mellitus hospitalized during 2009. 242 primary care physicians.	Retrospective correlational study. Physicians’ Jefferson Scale of Empathy scores were compared with occurrence of metabolic complications.	The odds ratio (OR) of 0.59, obtained by comparing low and highJSE scorers, indicates that shifting from a low- to a high-scoring category of physician empathy decreased the odds of metabolic complications among diabetic patients by 41%.
What to wear today? Effect of doctor’s attire on the trust and confidence of patients (Rehman *et al*., 2005)	Written survey after reviewing pictures of physicians in four different dress styles.	400 patients and visitors in the waiting room of an internal medicine outpatient clinic.	Cross-sectional descriptive study. Survey methodology.	Respondents significantly favored the professional attire with white coat (76.3%). Trust and confidence were significantly associated with their preference for professional dress.

## An incentive to find other studies

In this article we prioritized high-impact studies to cover a wide range of medical interventions and disciplines. It is not intended to be an exhaustive review of the literature. It should be seen as examples of studies that can be shared with colleagues and trainees. It should also act as an incentive to find other examples. To conduct this search in Pubmed, we used the detailed description of each role ( Frank et al., 2015). Among co-authors and with clinician educators of other subspecialties, we listed examples of clinical topics (e.g. antimicrobial stewardship) and outcomes (e.g. compliance) for each role. We encourage readers to use the list presented in Table 7 as a starting point to look for other studies more specific to their context or discipline.

**Table 7.** Clinical Topics and Outcomes Related to Six Intrinsic Roles Played by Physicians

**Physicians’ intrinsic roles according to the CanMEDS framework** ( Frank *et al.*, 2015)	**Non-exhaustive list of topics and outcomes related to each role to facilitate the literature search in clinical medicine**
**Collaborator** *As collaborators, physicians work effectively with other health care professionals to provide safe, high-quality, patient-centred care*	• Interprofessional work • Multidisciplinary team • Co-management • Teamwork or acute care team (e.g. resuscitation team) • Handover, handoff, patient transfer • Chart, consultation letters • Transition of care, patient referral • Ambulatory clinics • Chronic diseases clinics /management [...] board (e.g. tumor board, thyroid nodule board, glomerulonephritis board) • [Peri-]operative medicine
**Communicator** *As communicators, physicians form relationships with patients and their families that facilitate the gathering and sharing of essential information for effective health care*	• Patient [or parents, or families] experience • Patient education • Motivating approach • Informed consent • Shared decision-making • Patient partners • Advance directives for medical decisions, advance care planning • Substituted consent • Compliance • Patient discharge • Resource stewardship
**Scholar** *As scholars, physicians demonstrate a lifelong commitment to excellence in practice through continuous learning and by teaching others, evaluating evidence, and contributing to scholarship*	• Evidence-based medicine • Critical appraisal [of medical studies] • Continuous professional development [CPD] • Continuing medical education [CME] • Adherence to practice guidelines • [Individual or group] practice audits • Journal clubs • Grand rounds • Clinical teaching • Community of practice [in health care]
**Health Advocate** *As health advocates, physicians contribute their expertise and influence as they work with communities or patient populations to improve health. They work with those they serve to determine and understand needs, speak on behalf of others when required, and support the mobilization of resources to effect change*	• Preventive medicine, measures • Public health, campaign • Violence, stress, distress, trauma, accident prevention • Medical [or health professional] counselling • Exercise prescription • Nutritional [or dietary] advice (e.g. salt reduction) • Non-pharmacological advice • Illness (e.g. cancer) prevention, screening • Smoking, alcohol, drug weaning or cessation • Immunisation, vaccination • Stewardship (e.g. antimicrobial stewardship) • Prophylaxis
**Leader** *As leaders, physicians engage with others to contribute to a vision of a high-quality health care system and take responsibility for the delivery of excellent patient care through their activities as clinicians, administrators, scholars, or teachers*	• [Health care] management, organisation • [Human or material] resource allocation, stewardship • Quality improvement • Efficiency improvement • Patient safety • Service corridors • Team management, functioning • Stewardship • Opinion leaders • Health reforms • Practice change [Medication, procedures, investigation] protocols • [Health care related] Committee
**Professional** *As professionals, physicians are committed to the health and well-being of individual patients and society through ethical practice, high personal standards of behaviour, accountability to the profession and society, physician-led regulation, and maintenance of personal health*	• [Bio]ethical decisions, standards • Disadvantaged communities, patients, groups • Medical accountability, responsibility, commitment • Patients’ trust, confidence, respect, expectations • Empathy, transparency, honesty [of physician] • Consent • Confidentiality • Physician-assisted dying • Access to care • Discharge [or transfer] planning • Delays [or waiting list], cancellations, [lost to] follow up • [Physicians] staffing patterns, scheduling • Pursuit, lawsuit Coroner [or medical examiner] inquest

## Conclusion

After reflecting on these examples, clinicians and trainees should be convinced of the clinical impact of the abilities developed within each intrinsic role. Hopefully, clinicians will be able to recognize when an intrinsic role is at play in a clinical study and seize this teaching opportunity. A question will remain: do we have proof that teaching those roles to students will lead to those impressive clinical outcomes? Such studies, reaching the highest level of Kirkpatrick’s classification, remain exceptional in the medical education literature ( Kirkpatrick, 1994; Asch et al., 2014).

All thirty examples show that that the abilities of health professionals (individually or in a team) can be improved even after years of practice: clinicians starting to work together, others improving their handovers or adhering to recent guidelines. What is meant by intrinsic roles is the combination of multiple, sometimes subtle, ways that medicine is ideally practiced. Those abilities are more easily scaffolded during training than afterwards ( Arora et al., 2009; Whitehead et al., 2011; Steven et al., 2014; Renting et al., 2017). Indeed, the initial skills developed during training have a persistent impact on the overall quality of care that clinical experience may take years to catch up with ( Asch et al., 2014).

## Take Home Messages

• Trainees must develop clinical skills that combine their role of medical expert with the intrinsic roles of physicians (e.g. collaborator)
• Intrinsic roles are often misinterpreted and underused by supervisors in their everyday teaching habits
• Supervisors struggle in making the connection between clinical topics and intrinsic roles
• Although not worded in those terms, many clinical studies prove the impact of all the roles played by physicians on patient mortality, morbidity, readmission rate, or compliance
• Associating intrinsic roles with clinical indicators of performance can be an incentive for clinicians who usually focus their teaching and feedback on interventions with proven clinical impacts

## Notes On Contributors

**Dr Alexandre Lafleur, M.D., MSc(Ed.)** is Assistant Clinical Professor of medicine and Co-Chairholder of the QMA-CMA-MD Educational Leadership Chair in Health Professions Education at the Faculty of Medicine of Université Laval (Québec city, Canada). His research interests are post-graduate education and assessment.

**Dr Mathieu Gagné, M.D.** and **Dr Véronique Paquin, M.D.** are senior residents in internal medicine at Université Laval.

**Mrs Claudie Michaud-Couture** acted as research assistant for the QMA-CMA-MD Educational Leadership Chair in Health Professions Education.

## Declarations

The author has declared that there are no conflicts of interest.

## Ethics Statement

No involvement of students, patients. Article based on a literature review.

## External Funding

This article has not had any External Funding
