# Supplementary material for: How to Convince Clinicians that ‘Soft’ Skills Save Lives? Practical Tips to Use Clinical Studies to Teach Physicians’ Roles
Source: MedEdPublish (2016). 2019 May 30;8:119. [Version 1] doi: 10.15694/mep.2019.000119.1 (PMC10712525; doi:10.15694/mep.2019.000119.1)
Supplement: Peer review history

# Reviewer report: Reviewer response for version 1

**DOI:** 10.21956/mep.19848.r29942

**Published:** 2019-06-30

**Authors:** Debra Kiegaldie

**Affiliations:** 1 Holmesglen Institute & Healthscope Australia

This review has been migrated. The reviewer awarded 5 stars out of 5 This is an innovative, highly practical and very reader-friendly paper. It is almost a ‘one stop shop’ for examining the evidence about intrinsic roles and will be valuable for those who work in medical education. It clearly defines ‘intrinsic’ roles and provides excellent outcomes-based papers that highlight the impact of these roles. I particularly like the linkage to the CanMEDS framework. I just wonder about the choice of the word ‘prove’ as we know this is always challenging to categorically state. Maybe support? In collaborator, the authors state ‘interprofessional’ or ‘multidisciplinary’. This is the common terminology. There is an alternative way of viewing this which the authors may like to consider. Interprofessional infers collaboration between other health, (or even non health) professionals. Multidisciplinary infers collaboration between other disciplines but it is unclear whether this means within the profession or across professions as this term is often used erroneously in health care. I would like to suggest the term intraprofessional (rather than multidisciplinary) to highlight the equal importance that collaboration within a profession has in relation to intrinsic roles as well as collaboration between other professions (interprofessional). I realise it is semantics but getting the terminology right is important. Taking medicine as an example, that could be the collaboration between a physician, the surgeon, the geriatrician, the general practitioner to name a few. Most of your cited papers seem to mention intraprofessional. As this is an under-explored area, it might be useful to restate this to include this terminology and examine high impact studies that explore this (if any). To that effect, can your table please include the nature of the team members (doctor, nurse, allied health professional, surgeon. I am also surprised that the table doesn’t seem to include any interprofessional papers. I am sure there are some that may be worth including that have high impact outcomes.Overall, a very well written paper and one I strongly recommend.

Reviewer Expertise:

NA

No decision status is available

# Reviewer report: Reviewer response for version 1

**DOI:** 10.21956/mep.19848.r29944

**Published:** 2019-06-05

**Authors:** Balakrishnan Nair

**Affiliations:** 1 Centre for Medical Professional Development and University of Newcastle

This review has been migrated. The reviewer awarded 5 stars out of 5 This is an excellent paper. “Soft skills” are equally important to medical skills . They should be taught , nurtured and supported by all teachers. So this paper is timely; it is well researched and written. It is very educational for all teachers to note that these intrinsic skills have major positive outcome on patients ; they reduced morbidity and mortality as shown in this article. So we have no excuse not to teach and assess these skills I strongly recommend this paper

Reviewer Expertise:

NA

No decision status is available

# Reviewer report: Reviewer response for version 1

**DOI:** 10.21956/mep.19848.r29943

**Published:** 2019-06-04

**Authors:** Subha Ramani

**Affiliations:** 1 Harvard Medical School, Brigham and Women's Hospital

This review has been migrated. The reviewer awarded 5 stars out of 5 This is an excellent paper on the importance of the so called 'soft clinical skills', which I call the hardest clinical skills to excel in consistently. Very well written, well researched and referenced. The tips format works very well for this topic. The authors have done a fantastic job of summarizing literature under each of these skills and I think the term intrinsic skills is very appropriate. They have rightly emphasized the importance of these skills in CBME and their impact on patient care.Important topic for all clinical educators.

Reviewer Expertise:

NA

No decision status is available
